# Formononetin inhibits enterovirus 71 replication by regulating COX- 2/PGE_2_ expression

**DOI:** 10.1186/s12985-015-0264-x

**Published:** 2015-03-01

**Authors:** Huiqiang Wang, Dajun Zhang, Miao Ge, Zhuorong Li, Jiandong Jiang, Yuhuan Li

**Affiliations:** Institute of Medicinal Biotechnology, Chinese Academy of Medical Sciences and Peking Union Medical College, Beijing, 100050 China; Institute of Materia Medica, Chinese Academy of Medical Sciences and Peking Union Medical College, Beijing, 100050 China

**Keywords:** Enterovirus 71 (EV71), Formononetin, Antiviral activity, Drug development

## Abstract

**Background:**

The activation of ERK, p38 and JNK signal cascade in host cells has been demonstrated to up-regulate of enterovirus 71 (EV71)-induced cyclooxygenase-2 (COX-2)/ prostaglandins E_2_ (PGE_2_) expression which is essential for viral replication. So, we want to know whether a compound can inhibit EV71 infection by suppressing COX-2/PGE_2_ expression.

**Methods:**

The antiviral effect of formononetin was determined by cytopathic effect (CPE) assay and the time course assays. The influence of formononetin for EV71 replication was determined by immunofluorescence assay, western blotting assay and qRT-PCR assay. The mechanism of the antiviral activity of formononetin was determined by western blotting assay and ELISA assay.

**Results:**

Formononetin could reduce EV71 RNA and protein synthesis in a dose-dependent manner. The time course assays showed that formononetin displayed significant antiviral activity both before (24 or 12 h) and after (0–6 h) EV71 inoculation in SK-N-SH cells. Formononetin was also able to prevent EV71-induced cytopathic effect (CPE) and suppress the activation of ERK, p38 and JNK signal pathways. Furthermore, formononetin could suppress the EV71-induced COX-2/PGE_2_ expression. Also, formononetin exhibited similar antiviral activities against other members of *Picornaviridae* including coxsackievirus B2 (CVB2), coxsackievirus B3 (CVB3) and coxsackievirus B6 (CVB6).

**Conclusions:**

Formononetin could inhibit EV71-induced COX-2 expression and PGE_2_ production via MAPKs pathway including ERK, p38 and JNK. Formononetin exhibited antiviral activities against some members of *Picornaviridae*. These findings suggest that formononetin could be a potential lead or supplement for the development of new anti-EV71 agents in the future.

## Background

Enterovirus 71 (EV71) is a single-stranded, positive-sense RNA virus belonging to the enterovirus genus of the *Picornaviridae* family [1]. EV71 is a common enterovirus that usually causes hand, foot and mouth disease in children mostly under 5 years. Most EV71 infections result in mild clinical symptoms and are self-limiting, small part EV71 infections is often associated with neurological diseases include aseptic meningitis, brain stem encephalitis and acute flaccid paralysis. EV71 was first isolated from patients with central nervous system diseases in California in 1969. Since then, outbreaks have been periodically reported worldwide, especially in the Asia-Pacific region. Hundreds of cases involving lethal complications have been reported in each outbreak [2,3].

EV71 is neurotropic virus that can cause severe neurological diseases associated with inducing inflammation in the CNS region [4,5]. And, some researchs reported that EV71 infections were related to cyclooxygenase-2 (COX-2)/prostaglandins E_2_ (PGE_2_) pathway [6,7]. Cyclooxygenase (COX) is the rate-limiting step in the production of prostaglandins (PGs) from arachidonic acid. The acute encephalitis during virus or bacteria infection is partially mediated through cyclooxygenase-2 (COX-2)/prostaglandins (PGE_2_) expression. It had been reported that the expression of COX-2 could be observed during some viral infections including coxsackievirus B3 (CVB3) and theiler’s murine encephalomyelitis virus (TMEV) [8]. Studies have reported that COX-2 expression increased in human and rat nerve cell with EV71 infection through the action of MAPKs, NF-kB, and AP-1 and EV71-induced COX-2 expression and PGE_2_ production promoted viral replication via cAMP signaling [6,9]. Moreover, COX-2 expression and PGE_2_ generation increased by EV71 might be required for EV71 viral replication. So, targeting COX-2 and their upstream signaling components should yield useful therapeutic targets for EV71 intection.

EV71 could cause severe complications and even death in each outbreak. Unfortunately, currently there is no approved vaccine or antiviral drug for EV71-induced disease prevention and therapy. There is a clear need to develop inhibitors for EV71. There are some compounds reported exhibited antiviral activity against EV71, such as pleconaril [10], pyridyl imidazolidinones [11], Ganoderma lucidum triterpenoids [12], GuiQi [13], 7-Hydroxyflavone [14], chebulagic acid [15], chlorogenic acid [16], epigallocatechin gallate [17], chrysosplenetin [18], penduletin [18], 7-Hydroxyisoflavone [19], chrysin [20], pirodavir [21], and so on. However, it was just a simple evaluation of compounds as anti-EV71 agents and there were no in-depth mechanism researches to be done. So, there is an urgent need to develop an effective anti-EV71 agent for the control of EV71 infection.

**Figure 1:**
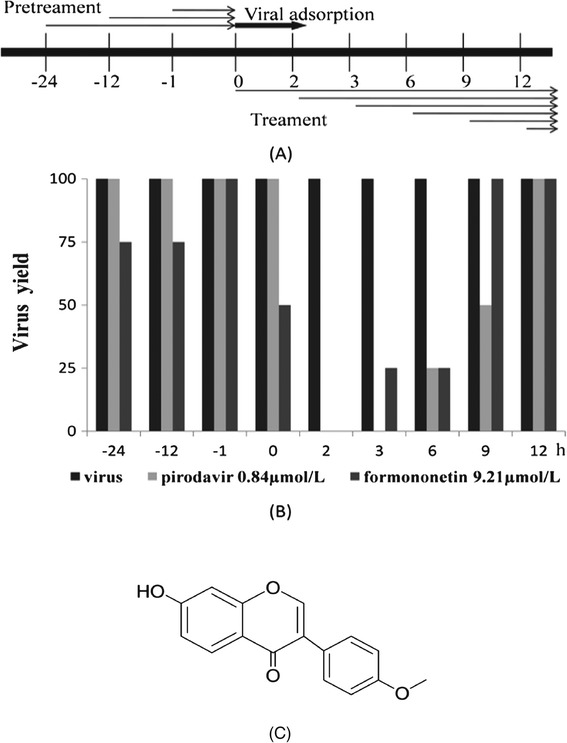
**The chemical structure of formononetin and time course assay. (A)** The illustration of the protocol of viral adsorption and treatment with formononetin and pirodavir. SK-N-SH cells were infected with 80 pfu/well of EV71 (SHZH98). Formononetin (9.21 μM) and pirodavir (0.84 μM) was added at the indicated times, respectively. **(B)** The morphological changes of EV71-infected cells treated with or without formononetin and pirodavir were observed at 48 h post-infection. The cytopathic effect (CPE) assay was shown. **(C)** The chemical structure of formononetin.

In a large-scale screening for novel candidates exhibiting activity against EV71, we found that formononetin was active against EV71 infection (Figure 1C).

Formononetin, a kind of plant isoflavonoids, was isolated from a number of plants and herbs such as the red clover. It has been reported to have multi pharmacological effects such as anti-inflammatory [3,22,23], antioxidant [24], wound healing [25], antitumor [26,27], vasorelaxant [28], cardioprotective activies [29] and neuroprotective activies [30] by regulating the mitogen-activated protein kinases (MAPKs) including ERK, p38, c-Jun-N terminal kinase (JNK). However, no antiviral activity has been reported for formononetin although some flavonoids and isoflavonoids reported exhibited anti-EV71 activities such as chrysin [20], 7-Hydroxyflavone [14], chrysosplenetin [18], penduletin [18] and 7-Hydroxyisoflavone [19]. Moreover, most compounds are just a simple evaluation effects as anti-EV71 agents and researches about the anti-EV71 mechanism of flavonoids and isoflavonoids had been reported rarely. Against this background, it would be interesting to know whether formononetin can influence the propagation of EV71 by inhibiting EV71-induced COX-2 expression and PGE_2_ production via MAPKs pathway including ERK, p38 and JNK. In this study, we evaluated the antiviral effect of formononetin on EV71 replication in Vero and SK-N-SH cells. We further explored the mechanism of the antiviral activity of formononetin against EV71. Our findings will shed new light on the design of anti-EV71 agents for HFMD patients especially those with neurological complications.

## Results

### Inhibition of EV71 replication by formononetin

Before detecting the antiviral effect of formononetin in vitro, we first detected the toxicity of formononetin in cells. As shown in Table 1, the 50% toxicity concentration (TC_50_) of formononetin was 149.38 μmol/L in Vero cells and 198.80 μmol/L in SK-N-SH cells through the MTS assay. The maximal non-toxicity dose (TC_0_) of formononetin was 41.42 μmol/L in Vero cells and 55.92 μmol/L in SK-N-SH cells through the MTS assay. The TC_50_ of pirodavir was 22.33 μmol/L in Vero cells and 30.30 μmol/L in SK-N-SH cells by MTS assay. The TC_0_ of pirodavir was 7.52 μmol/L in Vero cells and 10.83 μmol/L in SK-N-SH cells through the MTS assay.

**Table 1:** **The efficiency of formononetin and pirodavir against EV71 in vitro**

**Cell Compound**	**Vero**				**SK-N-SH**			
	**TC0 (μmol/L)**	**TC50 (μmol/L)**	**IC50(μmol/L)**	**SI**	**TC0 (μmol/L)**	**TC50 (μmol/L)**	**IC50 (μmol/L)**	**SI**
Formononetin	41.42 ± 0	149.38 ± 12.15	3.45 ± 0.55	43.30	55.92 ± 0	198.80 ± 6.02	3.98 ± 0.80	49.95
Pirodavir	7.52 ± 0	22.33 ± 1.71	0.38 ± 0.08	58.76	10.83 ± 0	30.30 ± 4.03	0.23 ± 0.04	131.74

Values provided in this table represent the mean of three independent experiments.

The IC_50_ of formononetin for EV71 SHZH98 strain was 3.45 μmol/L in Vero cells and 3.98 μmol/L in SK-N-SH cells. This was much lower than the TC_50_. The selectivity index (SI) is 38.11 in Vero cells and 43.07 in SK-N-SH cells (Table 1). The TC_50_, IC_50_ and SI of formononetin for other EV71 strains (JS-52, H and BrCr) in Vero cells are list in Table 2. These results demonstrated that formononetin can significantly inhibit the replication of EV71. Therefore, formononetin was a safe and effective agent against EV71.

**Table 2:** **The efficiency of formononetin against other EV71 strains and CVBs in vitro**

	**TC50 (μmol/L)**	**IC50 (μmol/L)**	**SI**
JS-52	149.38 ± 12.15	17.87 ± 8.51	78.36
H	149.38 ± 12.15	11.11 ± 9.23	13.45
BrCr	149.38 ± 12.15	6.47 ± 4.40	23.09
CVB2	149.38 ± 12.15	9.56 ± 2.23	15.63
CVB6	149.38 ± 12.15	5.33 ± 1.48	28.03
CVB3	149.38 ± 12.15	2.57 ± 0.87	58.12

Values provided in this table represent the mean of three independent experiments.

### Formononetin can reduce the RNA and protein synthesis of EV71

**Figure 2:**
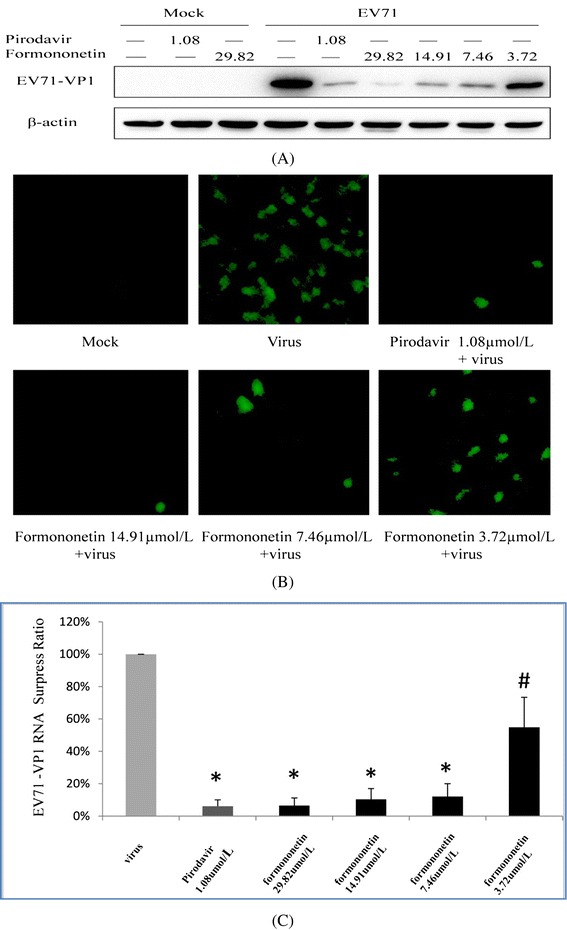
**The antiviral effect of formononetin and pirodavir against EV71 in SK-N-SH cells. (A)** Formononetin and pirodavir reduced the expression of EV71 VP1 protein in SK-N-SH cells by western blot assay. **(B)** Formononetin and pirodavir reduced the expression of EV71 VP1 protein in SK-N-SH cells by immunofluorescence assay. Cells were examined using a fluorescence microscopy (×100). **(C)** Formononetin and pirodavir reduced the expression of EV71 VP1 RNA by one-step qRT-PCR assay. *P < 0.001 ^*#*^ *P < 0.05*.

To examine the effect of formononetin on the biological synthesis of EV71, the EV71 (SHZH98 strain) infected cells was treated with formononetin and pirodavir and then the VP1 protein was analyzed by Western blotting. As shown in Figure 2A, formononetin can decrease the expression of VP1 protein in a dose dependent manner. The immunofluorescence assay also showed a similar result (Figure 2B). This result is consistent with the Western blot detection. Furthermore, formononetin treatment dose-dependently decreased the levels of EV71 RNA measured by reverse transcription-quantitative polymerase chain reaction (RT-qPCR) assay (Figure 2C). Pirodavir can also decrease the RNA and protein synthesis of EV71. These results suggested that both formononetin and pirodavir exhibit effectively anti-EV71 activity.

### Time course assay

Time course assay was performed to explore which stage formononetin and pirodavir function during EV71 replication. As shown in Figure 1B. We found that treatment with formononetin at 24 and 12 h before, and 0 to 6 h after EV71 infection was able to effectively reduce virus-induced CPE, while it did not exert this effect at −1, 9, and 12 h of EV71 infection. However, pirodavir treatment functioned only after viral infection (0–9 h p.i.) rather than before or during the infection.

### Formononetin can inhibit EV71-induced phosphorylation of ERK, p38 and JNK

**Figure 3:**
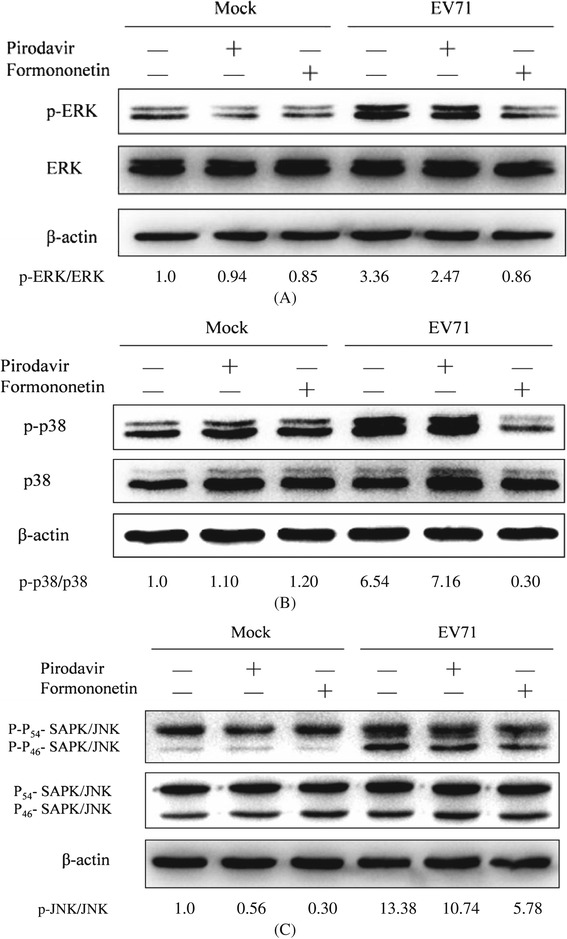
**Formononetin but not pirodavir reduces the phosphorylation of ERK (A), p38 (B), and JNK (C) induced by EV71 infection.** SK-N-SH cells (9 × 10^5^ cells/well) were plated into 6-well culture plates. The cells were then pretreated with formononetin (14.91 μmol/L) and pirodavir (1.08 μmol/L), respectively. After 24 h, the cells were mock-infected or infected with 4.8 × 10^5^ pfu/well of EV71 (SHZH98) for 15 min **(A)**, 10 min **(B)**, and 30 min **(C)**, respectively. The cells were harvested and ERK (A) p-ERK (A), p38 **(B)**, p-p38 (B), JNK **(C)**, and p-JNK **(C)** were detected by western blot.

It was known that ERK, p38 and JNK were downstream components of epidermal growth factor receptor (EGFR) signaling pathways which can be stimulated by several stimuli including EV71 infection in various cell types [31-34]. Thus, we assessed whether the EV71-induced phosphorylation of ERK, p38 and JNK could be reduced by formononetin. As shown in Figure 3, EV71-stimulated phosphorylation of ERK, p38 and JNK were significantly attenuated by treatment with formononetin (14.91 μmol/L) but not pirodavir.

### Formononetin can inhibit EV71-induced COX-2 expression and PGE_2_ generation

**Figure 4:**
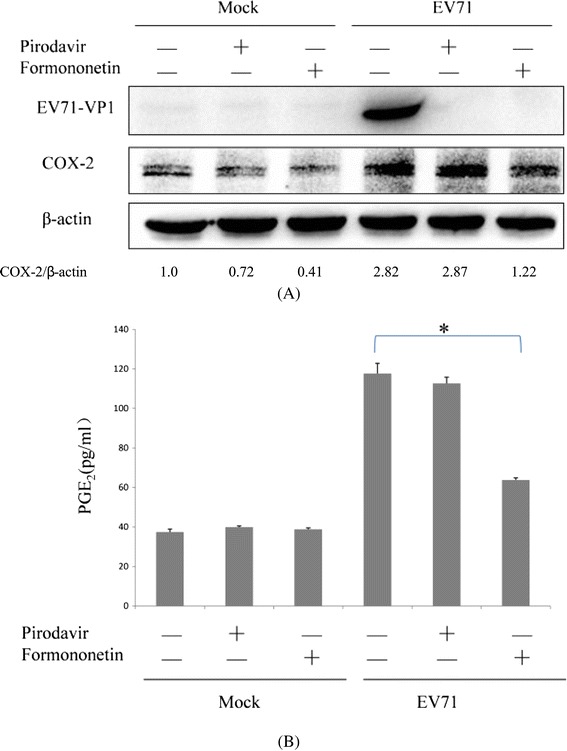
**Formononetin could inhibit EV71-induced COX-2 expression and PGE**_**2**_ **production. (A)** Formononetin but not pirodavir can reduce the expression of COX-2 induced by EV71 infection. SK-N-SH cells were mock-infected or infected with 480 pfu/well of EV71 (SHZH98) for 2 h. The cells were then treated with formononetin (14.91 μmol/L) and pirodavir (1.08 μmol/L), respectively, for 24 h. The cells were harvested and EV71-VP1 and COX-2 were examined by western blot. **(B)** Formononetin but not pirodavir can reduce the release of PGE_2_ stimulated by EV71 infection. SK-N-SH cells were pretreated with formononetin (14.91 μmol/L) or pirodavir (1.08 μmol/L) for 24 h. The cells were infected with 4.8 × 10^5^ pfu/well of EV71 (SHZH98). At 24 h p.i., the culture supernatants were harvested for PGE_2_ release assay.

It had been previously described that MAPKs were involved in COX-2 expression induced by EV71 infection [6]. We found that EV71-stimulated COX-2 expression in SK–N–SH cells was inhibited by pretreatment with formononetin (14.91 μmol/L) but not pirodavir (Figure 4A). PGE_2_ is a major product of COX-catalyzed arachidonic acid metabolism and the PGE_2_ level can act as a marker of functional activity of COX-2 expression. EV71-induced the expression of COX-2 was associated with PGE_2_ production in SK–N–SH cells [6]. Interestingly, EV71-stimulated PGE_2_ release in SK–N–SH cells was attenuated by pretreatment with formononetin but not pirodavir (Figure 4B). These results demonstrated that EV71-induced COX-2 expression and PGE_2_ release in SK–N–SH cells can been attenuated by formononetin but not pirodavir.

### Inhibition of CVBs replication by formononetin

Several isoflavonoids were found to exhibit broad-spectrum antiviral activity against picornaviruses. Thus, we speculated that formononetin might inhibit CVBs replication. The CPE reduction assay was used to determine the anti-CVBs activities of formononetin. Indeed, formononetin significantly inhibited the biosynthesis of CVB2, CVB6 and CVB3 in Vero cells. The IC_50_ and therapeutic index of formononetin for CVBs were showed in Table 2.

## Discussion

EV71, a member of the *Picornaviridae* family, causes HFMD by spreading through contact with virus-containing body fluids, respiratory droplets, and feces. However, there is no effective antiviral drug available for the treatment of HFMD. Some natural medicinal compounds have demonstrated to be active against the disease by ameliorating the symptoms and shortening the course [35,36]. The current absence of antiviral strategies for EV71 highlight the urgency of developing novel antiviral approaches.

Formononetin, a methoxylated isoflavone, is a main component in many traditional Chinese medicines such as Trifolium pratense, Radix Puerariae, Astragalus membranaceus, Lignum Dalbergiae Odoriferae, Suberect Spatholobus Stem and Radix hedysari. Previous studies have indicated that formononetin had many pharmacological properties such as antioxidative [24], anti-inflammatory [3,22,23], anticancer activities [26,27], acceleration of wound repair [25], neuronal protective [30] and cardioprotective effects [29]. As a phytoestrogen, formononetin also functions in menopause treatment. However, the antiviral activity of formononetin is rarely reported. In this study, we evaluated the anti-enterovirus activities of formononetin.

Picornaviruses are important human pathogens. Up to now, there is still no specific anti-picornavirus agent approved for clinical application. Three capsid-targeting molecules known as “WIN” compounds are currently in clinical development [37,38]. According to our results (dates not shown), the antiviral activity of pirodavir against EV71 is better than pleconaril. Thus, we used pirodavir as positive control for the evaluation of the antiviral activity of formononetin.

In the present study, we found that formononetin could inhibit the EV71-induced CPE in Vero or SK-N-SH cells. Formononetin could also inhibit viral RNA and protein synthesis in a dose-dependent manner. Time course assay in SK-N-SH cells showed that formononetin acted at an early stage of EV71 infection. The pretreatment with formononetin before infection also effectively inhibited EV71 replication. These findings demonstrated that formononetin exhibited strong antiviral activity against EV71. Moreover, formononetin showed antiviral activities against CVB2, CVB3 and CVB6. The preliminary analysis indicated that formononetin could inhibit EV71-induced COX-2 expression and PGE_2_ production via MAPKs pathway including ERK, p38 and JNK.

There are some flavonoids and isoflavonoids reported exhibited anti-EV71 activities such as chrysin [20], 7-Hydroxyflavone [14], chrysosplenetin [18], penduletin [18] and 7-Hydroxyisoflavone [19]. However, most compounds are just a simple evaluation effects as anti-EV71 agents and researchs about the anti-EV71 mechanism of flavonoids and isoflavonoids had been reported rarely. Our research is the first time to report the anti-EV71 mechanism of formononetin. Also, our research is the first time to find that flavonoids and isoflavonoids could inhibit EV71-induced COX-2 expression and PGE_2_ production via MAPKs pathway including ERK, p38 and JNK. Moreover, the selectivity indices (SI) of formononetin is similar to chrysosplenetin [18] and penduletin [18], and SI of formononetin is better than 7-Hydroxyisoflavone [19].

Overall, our findings provide a new clue for developing the anti-EV71 drug which acts on inhibition of both viral replication and inflammatory factors expression. This may offer hopes in the treatment of HFMD patients with CNS diseases. However, many questions remain, e.g., whether formononetin is effective antiviral therapy agains EV71 without adverse toxic effects in vivo and whether the proposed reduction in neurological complications is due to the reduced expression of COX2/PGE_2_. We will validate this approach in mouse models prior to clinical trials in further studies.

## Conclusion

In this study, we found that formononetin could inhibit EV71-induced COX-2 expression and PGE_2_ production via MAPKs pathway including ERK, p38 and JNK. Formononetin exhibited antiviral activities against some members of *Picornaviridae*. These findings suggest that formononetin could be a potential lead or supplement for the development of new anti-EV71 agent in the future.

## Materials and methods

### Cells and viruses

Vero cells were purchased from the American Type Culture Collection (ATCC), and were cultured in Minimum Essential Medium (MEM) supplemented with 10% fetal bovine serum (FBS) (GBICO) and antibiotics (100 U/ml penicillin and 100 mg/ml streptomycin). SK-N-SH cells were also purchased from the ATCC, and were cultured in Dulbecco’s Modified Eagle Medium: Nutrient Mixture F-12 (DMEM/F-12) supplemented with 10% FBS and antibiotics (100 U/ml penicillin G and 100 mg/ml streptomycin).

EV71 strain SHZH98 isolated from the throat swab sample of an HFMD case occurring in 1998 in China was kindly provided by Dr. Qi Jin, Institute of Pathogen Biology, Chinese Academy of Medical Science and Peking Union Medical School, Beijing, China. EV71 strain BrCr (VR-1775) and H (VR-1432) were purchased from the ATCC. EV71 strain JS-52 was a kind gift from Dr. Xiangzhong Ye, Beijing Wantai Biological Pharmacy Enterprise Co., Ltd. EV71 strain SHZH98 was passaged in Vero and SK-N-SH cells. EV71 strains BrCr, H, and JS-52 were passaged in Vero cells. CVB2 (strain Ohio-1), CVB3 (strain Nancy) and CVB6 (strain Schmitt) were all got from the ATCC and passaged in Vero cells.

### Compounds

Formononetin was purchased from the J&K Chemical Ltd. (Beijing, China) and the purity is no less than 99%. Pirodavir was kindly provided by Professor Junhai Xiao, Laboratory of Computer-Aided Drug Design & Discovery, Beijing Institute of Pharmacology & Toxicology, Beijing, China. Both formononetin and piradavir were dissolved into DMSO.

### Plaque assay for virus titer

Vero cells (3 × 10^5^ cells/well) were plated into 24-well plate and the cells were infected with 10-fold serially diluted virus suspension (200 μl/well). Suspension was diluted in MEM with 2% FBS. After absorption at 37°C with 5% CO_2_ for 2 h, the virus suspension was replaced with MEM containing 2% FBS and 1% methyl cellulose. The medium was removed at 96 h after infection. The cells were washed with phosphate-buffered saline (PBS), and then fixed and stained with 5% crystal violet at 37°C for 1 h. Finally, the crystal violet was washed out with water and heat-dried at 37°C. The plaques were counted and virus titer (pfu/ml) was calculated.

### Cytotoxicity assay

Cytotoxic effect of formononetin on Vero or SK-N-SH cells was assayed by MTS (promega) assay. Briefly, cells (3 × 10^4^ cells/well) were seeded into 96-well culture plates and were incubated overnight. Then, the medium was removed and different concentrations of formononetin were applied in triplicate. After 3 days’ incubation, the cytotoxicity of formononetin was determined by MTS assay. The TC_50_ was defined as the concentration that inhibits 50% cellular growth in comparison with the controls and the TC_0_ was defined as the maximal non-toxicity dose.

### CPE inhibition assay for anti-EV71

The anti-EV71 activity of formononetin was assayed by CPE inhibition method. Briefly, cells (3 × 10^4^ cells/well) were plated into 96-well culture plates for incubation of 24 h. The medium was then removed and cells were infected with EV71 of 80 pfu/well. Then, various concentrations of formononetin were supplemented for incubation of another 48 h. The IC_50_ defined as the minimal concentration of inhibitor required to inhibit 50% of CPE was determined by Reed & Muench method. The selectivity index (SI) was calculated as the ratio of TC_50_/IC_50_ [19].

### Western blot analysis

Cells were lysed in the M-PER mammalian protein extraction reagent (Thermo, Rockford, IL) containing halt protease inhibitor single-use cocktail (Thermo). The protein concentration was determined by the BCA reagents (Thermo). About 10 μg proteins were denatured and applied to sodium dodecyl sulfate-polyacrylamide gel electrophoresis (SDS-PAGE). The electrophoresis products were transferred to a polyvinyl idenefluoride (PVDF) film and PVDF membranes were then incubated at room temperature with specific primary antibody. After a standard washing, membranes were incubated with horse radish peroxidase (HRP)-labeled secondary antibody. The assay developed using a chemiluminescent substrate. The primary antibodies used in this study included antibodies against β-actin, p-p44/p42 MAPK, p44/p42 MAPK, p-p38 MAPK, p38 MAPK, p-JNK, JNK (Cell Signaling Technology), EV71 VP1 (Abnova) and COX-2 (Santa Cruz Biotechnology). The goat anti-rabbit and anti-mouse HRP-labeled antibodies were obtained from Cell Signaling Technology.

### Immunofluorescence assay

SK-N-SH cells grown on glass coverslips (Thermo) were infected with EV71 (SHZH98) of 160 pfu/well for 2 h. Then, various concentrations of formononetin were supplemented for incubation of another 24 h. After incubation, the culture medium was removed and the cells were washed and fixed. The cells were permeabilized in 0.5% Triton X-100 at room temperature for 15 min and blocked in PBS containing 1% BSA for 60 min at room temperature. Cells were then incubated with an anti-EV71 VP1 antibody (Abnova) at a dilution of 1: 500 for 2 h at room temperature. After washing three times with PBS, the samples were reacted with Alexa Fluor 488-labeled goat anti-mouse secondary antibody (Beyotime Institute of Biotechnology, China) for 1 h at room temperature. After washing with PBS, images were taken using a fluorescence microscope (Olympus, IX71) [39].

### Quantitative reverse-transcription polymerase chain reaction (qRT-PCR) quantification

SK-N-SH cells (9 × 10^5^cells/well) were plated into 6-well culture plates for incubation of 16 h. The medium was removed and cells were infected with EV71 (SHZH98) of 480 pfu/well. After 2 h, various concentrations of formononetin were supplemented for incubation of another 24 h. The total RNA of the infected cells was extracted using the RNeasy Mini kit (QIAGEN) according to the manufacturer’s instructions. The one-step qRT-PCR was performed with SuperScript III Platinum SYBR Green One-step RT PCR Kit (Invitrogen) using the ABI 7500 Fast Real-Time PCR system (Applied Biosystems). The mRNA expression of EV71 VP1 was detected with sense primer 5’-GCAGCCCAAAAGAACTTCAC-3’ and antisense primer 5’-ATTTCAGCAGCTTGGAGTGC-3’ targeting a conserved region of the VP1 gene [40]. The β-actin mRNA was detected using sense primers 5’-TGACGGGGT CACCCACA CTGTGCCCATCTA-3’ and antisense primer 5’-CTAGAAGCATTTG CGGTGGACG ATG-3’.

### Time course assay

The antiviral activity of formononetin was examined at different time periods before and after viral infection as shown in Figure 1A. Briefly, cells (3 × 10^4^ cells/well) were seeded and incubated for 16 h. The cell monolayer was then infected with EV71 of 80 pfu/well. Different concentrations of formononetin were supplemented into cells following the protocol. Cells were then incubated for another 48 h and assayed with CPE assay.

### Measurement of PGE_2_ release

For the detection of PGE_2_ release, SK–N–SH cells (3 × 10^5^ cells/well) were seeded into 24-well culture plates and were incubated for 16 h. And then the cells were serum-deprived for 24 h and incubated with EV71 for the indicated time intervals. When drugs were used, they were added 24 h prior to the application of EV71. After treatment, the supernatants were harvested. The supernatant from each sample was assayed using a PGE_2_ EIA kit (AMEKO) following the manufacturer’s instruction. Briefly, we added sample, and successively anti-PGE_2_ antibody, streptavidin-HRP to the plate. The plate was closed with closure plate membrane and incubated for 60 min at 37°C. After uncovering closure plate membrane, liquid was discarded and the plate was washed with washing buffer for 5 times. The chromogen solution A and B were added to each well and incubated for 10 min at 37°C. The stop solution was then added to each well to stop the reaction. The absorbance of each well was measured at 450 nm [6].

### CPE inhibition assay for anti-CVBs

The anti-virus activity of formononetin on CVBs was also assayed by CPE method. Briefly, cells (3 × 10^4^ cells/well) were plated into 96-well culture plates for incubation of 24 h. The medium was removed and cells were infected with 100 TCID_50_ of CVB2, CVB6 or CVB3. Then, various concentrations of formononetin were supplemented for incubation of another 48 h. The IC_50_ was determined by Reed and Muench method. The SI was calculated as the ratio of TC_50_/IC_50_ [19].

## Abbreviations

EV71: Enterovirus 71
CNS: Central nervous system
CPE: Cytopathic effect
HFMD: Hand foot and mouth disease
TC_0_: The maximal non-toxicity dose
TC_50_: 50% toxicity concentration
IC_50_: 50% inhibitory concentration
SI: Selectivity index

## References

[1] Solomon T, Lewthwaite P, Perera D, Cardosa MJ, McMinn P, Ooi MH (2010). Virology, epidemiology, pathogenesis, and control of enterovirus 71. Lancet Infect Dis.

[2] McMinn PC (2012). Recent advances in the molecular epidemiology and control of human enterovirus 71 infection. Curr Opin Virol.

[3] Wang X, Zhu C, Bao W, Zhao K, Niu J, Yu XF (2012). Characterization of full-length enterovirus 71 strains from severe and mild disease patients in northeastern China. PLoS One.

[4] Weng KF, Chen LL, Huang PN, Shih SR (2010). Neural pathogenesis of enterovirus 71 infection. Microbes Infect.

[5] Wang SM, Liu CC (2009). Enterovirus 71: epidemiology, pathogenesis and management. Expert Rev Anti Infect Ther.

[6] Tung WH, Hsieh HL, Yang CM (2010). Enterovirus 71 induces COX-2 expression via MAPKs, NF-kappaB, and AP-1 in SK-N-SH cells: Role of PGE(2) in viral replication. Cell Signal.

[7] Tung WH, Hsieh HL, Lee IT, Yang CM (2011). Enterovirus 71 modulates a COX-2/PGE2/cAMP-dependent viral replication in human neuroblastoma cells: role of the c-Src/EGFR/p42/p44 MAPK/CREB signaling pathway. J Cell Biochem.

[8] Steer SA, Corbett JA (2003). The role and regulation of COX-2 during viral infection. Viral Immunol.

[9] Tung WH, Lee IT, Hsieh HL, Yang CM (2010). EV71 induces COX-2 expression via c-Src/PDGFR/PI3K/Akt/p42/p44 MAPK/AP-1 and NF-kappaB in rat brain astrocytes. J Cell Physiol.

[10] Zhang G, Zhou F, Gu B, Ding C, Feng D, Xie F (2012). In vitro and in vivo evaluation of ribavirin and pleconarilantiviral activity against enterovirus 71 infection. Arch Virol.

[11] Chen TC, Liu SC, Huang PN, Chang HY, Chern JH, Shih SR (2008). Antiviral activity of pyridyl imidazolidinones against enterovirus 71 variants. J Biomed Sci.

[12] Zhang W, Tao J, Yang X, Yang Z, Zhang L, Liu H (2014). Antiviral effects of two Ganoderma lucidum triterpenoids against enterovirus 71 infection. Biochem Biophys Res Commun.

[13] Pu X, Wang H, Li Y, Fan W, Yu S (2013). Antiviral activity of GuiQi polysaccharides against enterovirus 71 in vitro. Virol Sin.

[14] Wang J, Su H, Zhang T, Du J, Cui S, Yang F (2014). Inhibition of Enterovirus 71 replication by 7-hydroxyflavone and diisopropyl-flavon7-yl Phosphate. PLoS One.

[15] Yang Y, Xiu J, Liu J, Zhang L, Li X, Xu Y (2013). Chebulagic acid, a hydrolyzable tannin, exhibited antiviral activity in vitro and in vivo against human enterovirus 71. Int J Mol Sci.

[16] Li X, Liu Y, Hou X, Peng H, Zhang L, Jiang Q (2013). Chlorogenic acid inhibits the replication and viability of enterovirus 71 in vitro. PLoS One.

[17] Ho HY, Cheng ML, Weng SF, Leu YL, Chiu DT (2009). Antiviral effect of epigallocatechingallate on enterovirus 71. J Agric Food Chem.

[18] Zhu QC, Wang Y, Liu YP, Zhang RQ, Li X, Su WH (2011). Inhibition of enterovirus 71 replication by chrysosplenetin and penduletin. Eur J Pharm Sci.

[19] Wang HQ, Meng S, Li ZR, Peng ZG, Han YX, Guo SS (2013). The antiviral effect of 7-hydroxyisoflavone against Enterovirus 71 in vitro. J Asian Nat Prod Res.

[20] Wang J, Zhang T, Du J (2014). Anti-enterovirus 71 effects of chrysin and its phosphate ester. PLoS One.

[21] Barnard DL, Hubbard VD, Smee DF, Sidwell RW, Watson KG, Tucker SP (2004). In vitro activity of expanded-spectrum pyridazinyl oxime ethers related to pirodavir: novel capsid-binding inhibitors with potent antipicornavirus activity. Antimicrob Agents Chemother.

[22] Lai PK, Chan JY, Cheng L, Lau CP, Han SQ, Leung PC (2013). Isolation of anti-inflammatory fractions and compounds from the root of Astragalus membranaceus. Phytother Res.

[23] Krenn L, Paper DH (2009). Inhibition of angiogenesis and inflammation by an extract of red clover Trifolium pratense L.). Phytomedicine.

[24] Mu H, Bai YH, Wang ST, Zhu ZM, Zhang YW (2009). Research on antioxidant effects and estrogenic effect of formononetin from Trifolium pratense (red clover). Phytomedicine.

[25] Huh JE, Nam DW, Baek YH, Kang JW, Park DS, Choi DY (2011). Formononetin accelerates wound repair by the regulation of early growth response factor-1 transcription factor through the phosphorylation of the ERK and p38 MAPK pathways. Int Immunopharmacol.

[26] Auyeung KK, Law PC, Ko JK (2012). Novel anti-angiogenic effects of formononetin in human colon cancer cells and tumor xenograft. Oncol Rep.

[27] Chen J, Zeng J, Xin M, Huang W, Chen X (2011). Formononetin induces cell cycle arrest of human breast cancer cells via IGF1/PI3K/Akt pathways in vitro and in vivo. Horm Metab Res.

[28] Sun T, Liu R, Cao YX (2011). Vasorelaxant and antihypertensive effects of formononetin through endothelium-dependent and -independent mechanisms. Acta Pharmacol Sin.

[29] Zhang S, Tang X, Tian J, Li C, Zhang G, Jiang W (2011). Cardioprotective effect of sulphonated formononetin on acute myocardial infarction in rats. Basic Clin Pharmacol Toxicol.

[30] Sun M, Zhou T, Zhou L, Q C, Yu Y, Yang H (2012). Formononetin protects neurons against hypoxia-induced cytotoxicity through upregulation of ADAM10 and sAβPPα. J Alzheimers Dis.

[31] Kapoor GS, O’Rourke DM (2003). Receptor tyrosine kinase signaling in gliomagenesis: pathobiology and therapeutic approaches. Cancer Biol Ther.

[32] Ohtsu H, Dempsey PJ, Eguchi S (2006). ADAMs as mediators of EGF receptor transactivation by G protein-coupled receptors. Am J Physiol Cell Physiol.

[33] Fischer B, Marinov M, Arcaro A (2007). Targeting receptor tyrosine kinase signalling in small cell lung cancer (SCLC): what have we learned so far?. Cancer Treat Rev.

[34] Rodemann HP, Dittmann K, Toulany M (2007). Radiation-induced EGFR-signaling and control of DNA-damage repair. Int J Radiat Biol.

[35] Wei YH, Fang W, Wan ZY, Wang KM, Yang QY, Cai XF (2014). Antiviral effects against EV71 of pimprinine and its derivatives isolated from Streptomyces sp. Virol J.

[36] Chen X, Wang C, Xu L, Wang W, Yang G, Tan RX (2013). A laboratory evaluation of medicinal herbs used in china for the treatment of hand, foot, and mouth disease. Evid Based Complement Alternat Med.

[37] Clercq ED. Highlights in Antiviral Drug Research: Antivirals at the Horizon, Med. Res. Rev., 00, No. 0, 1–34, 2012 10.1002/med.21256 PMC7168470 22553111

[38] Thibaut HJ, De Palma AM, Neyts J (2012). Combating enterovirus replication: state-of-the-art on antiviral research. Biochem Pharmacol.

[39] Lin JY, Li ML, Huang PN, Chien KY, Horng JT, Shih SR (2008). Heterogeneous nuclear ribonuclear protein K interacts with the enterovirus 71 5’ untranslated region and participates in virus replication. J Gen Virol.

[40] Yi L, He Y, Chen Y, Kung HF, He ML (2011). Potent inhibition of human enterovirus 71 replication by type I interferon subtypes. Antivir Ther.

